# Paramedics’ Behavior Patterns When Transferring Non-Mobile Patients from the Ground to a Stretcher

**DOI:** 10.3390/healthcare13060611

**Published:** 2025-03-12

**Authors:** Maïté Tanguay, Jason Bouffard, Jasmin Vallée-Marcotte, Philippe Corbeil

**Affiliations:** 1 Department of Kinesiology, Faculty of Medicine, Université Laval, Quebec, QC G1V 0A6, Canada; maite.tanguay.1@ulaval.ca (M.T.); jason.bouffard@kin.ulaval.ca (J.B.); jasmin.vallee-marcotte.1@ulaval.ca (J.V.-M.); 2 Centre for Interdisciplinary Research in Rehabilitation and Social Integration (CIRRIS), Centre Intégré Universitaire de Santé et de Services Sociaux de la Capitale-Nationale (CIUSSS-CN), Quebec, QC G1V 0A6, Canada

**Keywords:** patient transfer, lifting, emergency medical service (EMS), know-how, competence, musculoskeletal disorders, field data

## Abstract

**Background/Objectives:** Transferring non-mobile patients from the ground to a stretcher represents one of the riskiest tasks for musculoskeletal disorders among emergency medical technicians–paramedics (EMT-Ps), but there is little information available on how they perform in real-life work situations. **Methods:** This study aimed to describe EMT-Ps’ patterns of behavior observed from field data and highlight safe work operations. A secondary analysis was conducted on 27 videos collected during EMT-Ps’ responses to live calls. Contextual variables (workspace and external assistance), operations during the preparation subtask (move patient or interfering objects and adjust stretcher’s height and position), and movements and postures related to the transfer subtask were extracted from the videos. **Results:** The results demonstrate that despite stratification based on similar contextual factors (equipment and limited workspace), EMT-Ps’ behavior varied between interventions during the preparation and transfer subtasks. Several operations to adjust the patient–stretcher configuration before the lifting phase were carried out to facilitate patient transfer, but these were not always optimal from a safety perspective. Strategies such as fast loading (1 out of 4) and the use of external assistance (6 out of 15) were beneficial in certain circumstances. **Conclusions:** EMT-Ps demonstrated their ability to analyze the situation, organize accordingly, and adapt their behavior by applying these safety skills.

## 1. Introduction

According to the U.S. Bureau of Labor Statistics, emergency medical technicians and paramedics (EMT-Ps) experienced 15,590 nonfatal occupational injuries and illnesses involving days away from work in 2021–2022 [1]. Of this number, 3590 were musculoskeletal disorders (MSDs) potentially caused by, among other things, overexertion and bodily reaction (29.1%) and falls, slips, or trips (7.6%). The body parts mainly affected were the back (14.1%), the knee (4.8%), and the shoulder (4.1%). EMT-Ps have a rate of work-related MSDs that is about three times the national average in the United States for all occupations [2] and have one of the highest rates of compensation claims among healthcare workers [3,4].

Adopting awkward work postures, maintaining these postures, and using excessive force constitute work-related factors that present a great risk for MSDs among EMT-Ps [5]. According to a field study, between 16% and 29% of a work shift is generally spent in harmful positions. Environmental, social, organizational, and contextual factors influence an EMT-P’s behavior, choices, and responses in specific situations [5]. Among contextual variables, a patient’s characteristics (body weight, size, and shape) and medical condition affect the EMT-P’s exposure to high-demand work activities linked to MSD risks [6,7,8].

EMT-Ps’ working environments are extremely varied and often confined or restrictive [3,5,7,8], which adds to the physical stress and difficulties of assisting a patient. Restricted spaces (e.g., cases where the patient is on the floor between the toilet and the bathtub, the toilet and the wall, or the bed and the wall or in a cluttered house) may also limit the type of external assistance and the use of assistive devices to help transfer the patient [8,9]. The results of a focus group conducted with EMT-Ps indicated that the participants often needed extra assistance to handle heavy patients, but a lack of space for additional providers may limit the amount of assistance that can be provided [9]. Based on the observation of real-life work activities during 175 EMT-P work shifts, Corbeil et al. [5] showed that the need for spinal immobilization (which affects the choice of assistive devices to help lift and move patients), a limited work area, and the use of external assistance have a substantial impact on risk exposure when transferring a non-mobile patient from the ground or floor to a stretcher.

Patient transfers (e.g., from the floor to a stretcher) in which the patient is non-mobile and cannot otherwise assist the EMT-Ps involve actions such as pushing, pulling, lifting, lowering, and potentially carrying a heavy person that increase their exposure to risk factors [5,10]. Larouche et al. [11] developed an overall risk index (ORI) to quantify the risk associated with non-mobile patient transfers in real-life work situations and compare the risks of families of transfers: patient lying above the ground (e.g., on a bed), sitting above the ground, or sitting or lying on the ground. The results conclusively suggested that patient transfers from the ground are the riskiest. ORI scores were greatly influenced by the patient’s weight and the EMT-Ps’ lifting styles (often influenced by contextual variables such as the work area), external assistance to help the EMT-Ps with the lifting task, and the EMT-Ps’ individual capacities, which influence perceived exertion. These findings are consistent with those derived from laboratory analysis, which showed that the greatest spine loads predicted by biomechanical analyses of simulated EMT-P tasks occurred when lifting a patient from the floor on a backboard [10]. Also, lifting patient actors who weighed 91 to 103 kg from the floor or from a recliner to a standing position led to significant spinal loading (peak L5/S1 anterior shear loads and L4/L5 spine compression force) [8] above the limits advocated in the ergonomic literature [12,13]. While the ORI is helpful for identifying particularly risky working contexts, it does not allow for a comprehensive understanding of task constraints and EMT-Ps’ behaviors, choices, and responses that mitigate or increase risks within specific situations (know-how).

Movements and postures influence back loading when lifting an object or a person; these include trunk flexion, starting position, foot stance, asymmetry, lever arm, etc. [14]. In manual handling, certain safety-related know-how is well documented, such as foot orientation for a faster transition [15] or greater knee flexion and less lumbar flexion when lifting [16]. Experts are known to execute preparatory tasks associated with safe practices more frequently than novices, such as orienting or bringing the load closer to the deposit zone before the lift [16]. In addition to posture and movement competences, material handlers use competences such as finding ways to alleviate task constraints; making decisions based on possible compromises between work performance, personal preferences, and health and safety issues; and acquiring information that guides their choices [17]. On the whole, the workers are not simply perceived as individuals who apply good working methods but rather as decision makers capable of solving problems by adapting their motor responses to the characteristics of the task [16,17,18].

Arial et al. [19] identified several working strategies during prehospital interventions that were believed to be intended to prevent back problems through posture stabilization mechanisms or by sharing the physical demand between colleagues. Workers’ safety know-how has been identified in work contexts, such as transferring a patient from a stair-chair to a stretcher [20] and loading a stretcher into an ambulance [21], but not yet for transfers of non-mobile patients from the ground to a stretcher. As laboratory studies have shown, some EMT-Ps may choose a lifting style that minimizes their biomechanical exposure while repeating a backboard lift from the ground, such as adopting a squat-like posture with minimal trunk flexion at the initiation of the lift [22]. Moreover, EMT-Ps have to adapt their working technique to the patient’s clinical condition and the specific equipment available. Several transfer devices that could potentially reduce the biomechanical loads experienced by EMT-Ps can be used to help lift a patient from the ground to the stretcher or from a lying to a sitting position or to ease lateral patient transfers [7,8,10,23,24,25,26]. The use of these different types of equipment requires preparatory steps incorporated into a sequence of operations that are often overlooked in standardized laboratory studies. A better understanding of EMT-Ps’ movements and postures in these high-risk work contexts is a necessary step in attempting to prevent MSDs in current and future workers.

This study’s two objectives were to describe patterns of EMT-Ps’ behaviors observed from field data and stratified by situational factors when transferring a non-mobile patient from the ground or floor to a stretcher and to highlight safe work operations during these patient transfers.

Based on the above findings, we propose, in this study, the consideration of the presence or absence of restrictive work environments for EMT-Ps, the type of external assistance available, and the use of assistive devices to aid non-mobile patient transfer as situational factors. To our knowledge, this study is the first to describe how EMT-Ps perform in real-life work situations when transferring non-mobile patients from the ground or floor to a stretcher. The novel element of this study resides in its understanding of how EMT-Ps can adapt to the variability of real prehospital work contexts associated with patient transfers.

## 2. Material and Methods

### 2.1. Field Data

This study reports on a secondary analysis of video data obtained while EMT-Ps responded to live calls in the areas of Quebec and Montreal (Canada) between 2011 and 2013. EMT-P candidates enrolled in the original study were required not to have missed work due to injury in the 30 days prior to enrollment. The analysis is based on a subset of 27 calls (out of 639 calls recorded on video) that required a non-mobile patient to be transferred from the ground or floor to a stretcher. The institutional Ethics Committee approved the initial project (approval number: 2010-151; approved on 19 July 2010) and authorized access to the database for this study (approval number: 2021-041; approved on 16 March 2021).

The video data focus mainly on one of the two EMT-Ps, emphasizing as much as possible the movements and postures of the whole body during each task performed. In addition to video data, additional information characterizing each prehospital intervention following the call was made available in the database (e.g., medical protocol, patient’s weight, and questionnaires).

**Figure 1:**
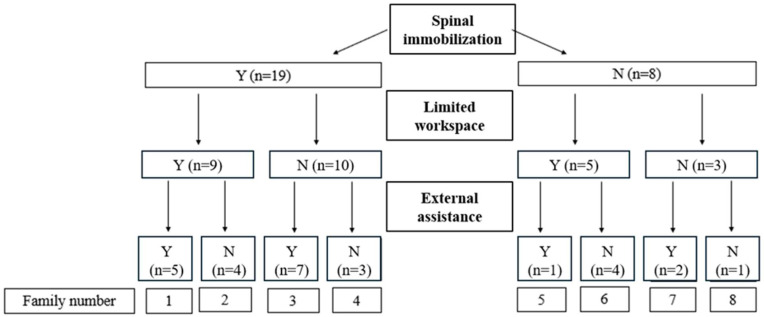
Classification used to stratify the transfer of non-mobile patients from the ground/floor to a stretcher according to work context. Y = yes; N = no; n = number of cases.

Because the work context in which the EMT-Ps intervene varies greatly from one call to another, three situational factors were used to stratify patient transfer tasks into eight families (Figure 1). These three situational factors were spinal immobilization, a limited work area, and the use of external assistance [5]. Each patient transfer family contains between one and seven cases.

### 2.2. Definition of a Non-Mobile Patient Transfer

**Figure 2:**
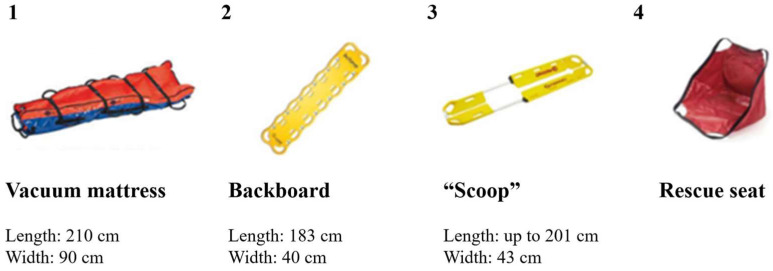
Illustration of four transfer devices for patient transfers and their specifications (which may vary depending on the manufacturer): (**1**) vacuum mattress, (**2**) backboard, (**3**) scoop, and (**4**) rescue seat. A vacuum mattress is the equipment prescribed for spinal immobilization of a patient with a traumatic injury. A backboard is an extraction or evacuation device and is not prescribed for immobilization unless the patient does not fit in the vacuum mattress. It can also be used with a patient in cardiac arrest. With the vacuum mattress, each EMT-P grabs two handles, often the ones located near the patient’s shoulder and pelvis. For the backboard and scoop, the EMT-Ps are located at each end and grab the equipment’s frame directly. For the rescue seat, each EMT-P grabs two handles (one located near the patient’s shoulder and the other under the patient’s thigh close to the knee).

According to Larouche et al. [11], the task of transferring a non-mobile patient starts with the first visual contact between the EMT-Ps and the patient and ends when the patient is properly positioned on the stretcher. The task is separated into three subtasks: preparation, transfer, and repositioning. During the preparation subtask, the EMT-Ps may execute several operations to prepare for an optimal transfer maneuver: secure the equipment, position the patient, make room by moving objects or furniture located in the working space, etc. The patient is then installed on the appropriate prescribed equipment, in accordance with the medical protocol in place (Figure 2). Most patient transfers necessitating spinal immobilization (families 1 to 4, Figure 1) were performed using a vacuum mattress (84.2%), while 15.8% were transferred using a backboard. When no spinal immobilization was required, EMT-Ps mainly used the patient’s clothing or the “scoop” (38% each), as well as the rescue seat or sheet (12% each), to transfer the patient (Figure 2).

The subtask following preparation is transfer, which consists of three phases: pickup, travel, and loading [11,27]. The pickup (“origin”) occurs immediately before the patient is moved (travel), and the operation ends with the loading onto the “destination” (stretcher). The transfer subtask is usually performed by two EMT-Ps, sometimes with external assistance. The transfer can be followed, if necessary, by the repositioning subtask, during which the patient is properly positioned or repositioned on the stretcher.

### 2.3. Ergonomic Observation Grids

Observation grids were designed to describe EMT-Ps’ behaviors and choices during the preparation and transfer subtasks. The repositioning subtask was not analyzed because it was observed only infrequently, and the video quality did not allow for the precise assessment of the small movements this subtask requires. The grids were developed in three steps: construction, validation, and grid use and item reduction.

#### 2.3.1. Grid Construction

The construction of the grids was necessary to help the observer analyze the video sequences and highlight the following elements: operations executed by the EMT-Ps during the preparation subtask, EMT-Ps’ movements and postures during the transfer subtask, and certain contextual variables observed during the preparation and transfer tasks (work area, type of grip, external assistance, and quality of external assistance).

The team first created an initial list of observable elements by observing a subset of interventions. The choice of items was refined iteratively following discussions between the members of the team. Consensus on the list of observable items was reached when all items were appropriate for all patient transfer families.

For the operations of the preparation subtask, favorable or unfavorable aspects were judged in relation to the health and safety dimension of EMT-Ps’ work. The literature tends to suggest that the most valid and best-discriminating rating systems contain six or more response categories or scale points [28]. A six-level categorization system was therefore chosen to rate the MSD risk associated with each operation (very unfavorable, unfavorable, neutral, favorable, very favorable, and not applicable or impossible to observe).

#### 2.3.2. Grid Validation

The inter-and intra-rater reliability of the grid were assessed. The first rater was a kinesiologist and an EMT-P since May 2012 (not working for either of the companies at the time the videos were shot) who led the construction of the grid. The second rater was an ergonomist who had experience with this type of grid. A 1 h training session was given on the specific terms used for the operations, postures and movements, and rating system used for the grid, followed by the observation and analysis of two videos for practice to ensure a common understanding of the observation and rating process (approximately 30 min). Both raters separately watched and analyzed 10 randomly selected interventions to evaluate inter-rater reliability. One month later, the first rater watched the same ten videos to evaluate intra-rater reliability. Percentages of agreement and Gwet’s AC1 measures were calculated to assess the intra- and inter-rater reliability of the 23 elements of the evaluation grid [29].

#### 2.3.3. Grid Use and Item Reduction

MT evaluated each of the 27 patient transfers using the grid. Ergonomic observations were used to highlight variations between patient transfer families and associated operations of the preparation subtask, along with their risks. The elements with an unfavorable/very unfavorable observation rate of 20% and lower were judged as non-significant and were not analyzed. Elements with a high proportion (≥20%) of non-observable ratings were also eliminated.

### 2.4. Data Synthesis

A description of the EMT-P behavior patterns of four patient transfer groupings (first two levels of stratification in Figure 1) is presented in the Results Section to highlight the interaction between the equipment use (vacuum mattress vs. other equipment, Figure 2) and the available workspace (limited vs. free workspace). The impact of assistance from bystanders was analyzed by considering all families with external assistance to extract the main trends. The duration of each transfer was calculated to assess the impact of contextual factors. Event-recording software (Observer™ XT 12, Noldus Information Technology BV, Wageningen, The Netherlands) was used to facilitate the identification of the beginning of the pickup phase and the end of the loading phase by the user.

## 3. Results

### 3.1. Ergonomic Observation Grids

**Figure 3:**
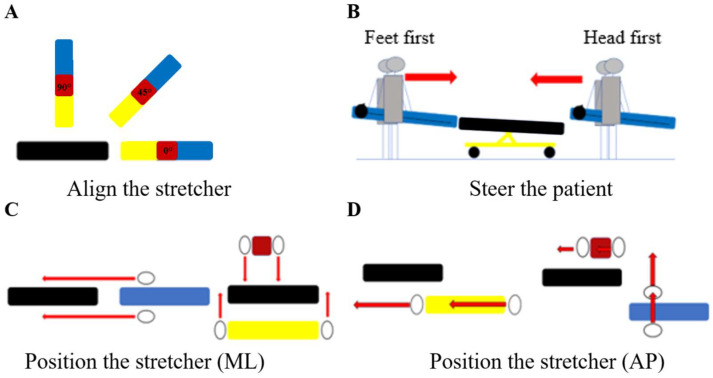
Subset of operations related to the patient–stretcher configuration observed during the preparation task involving the vacuum mattress (blue), scoop and backboard (yellow), or rescue seat (red): (**A**) “align the stretcher” refers to the angle between the two pieces of equipment in the horizontal plane; (**B**) “steer the patient” refers to the patient’s body part that is directed toward the stretcher first during the patient transfer task (“feet first” or “head first”); (**C**) position the stretcher in the medial–lateral (ML) direction according to the transfer equipment; (**D**) position the stretcher in the anterior–posterior (AP) direction. Note that EMT-Ps (illustrated by oval shapes on panels (**C**,**D**)) are positioned face to face when using the transfer equipment. The stretcher is shown in black and the red arrows indicate the direction of movement of the EMT-Ps.

The observable elements (n = 23) were separated into three categories: contextual variables (n = 3) (work area during transfer, external assistance, and quality of external assistance; Supplementary Table S1); operations during the preparation subtask (n = 7) (move interfering objects, move the patient, adjust the stretcher’s height, position the stretcher close to the patient (or vice versa) at an optimal lateral distance and an optimal anterior-posterior (AP) distance (separate operations), align the stretcher, and steer the patient; see Figure 3 and Supplementary Table S2); and movements and postures related to the transfer operation (n = 13; Supplementary Table S3). Movements and postures were separated according to the three phases (pickup, travel, and loading): EMT-Ps’ whole body positions (pickup and loading), postural asymmetry (pickup, travel, and loading), position of feet (pickup and loading), vertical hand position at pickup, lever arm (travel and loading), synchronized movements (pickup and loading), and loading methods. The mean and median percentages of intra-rater reliability were 0.81 and 0.80; the mean and median percentages of inter-rater reliability were 0.70 and 0.70 (Supplementary Table S4). Of the 23 elements included in the grids, only the foot position during loading was eliminated because the rate of non-observable items was 48.1%.

### 3.2. Description of EMT-Ps’ Behavior During Non-Mobile Patient Transfers

The mean duration of all transfer subtasks was 6.26 s (±2.69 s, median: 5.75 s). The transfers that required the longest time to complete belonged to the spinal immobilization and limited work area family (11.3 s and 13.2 s), and the spinal immobilization and suitable work area family (12.9 s).

#### 3.2.1. Spinal Immobilization and Limited Work Area

**Figure 4:**
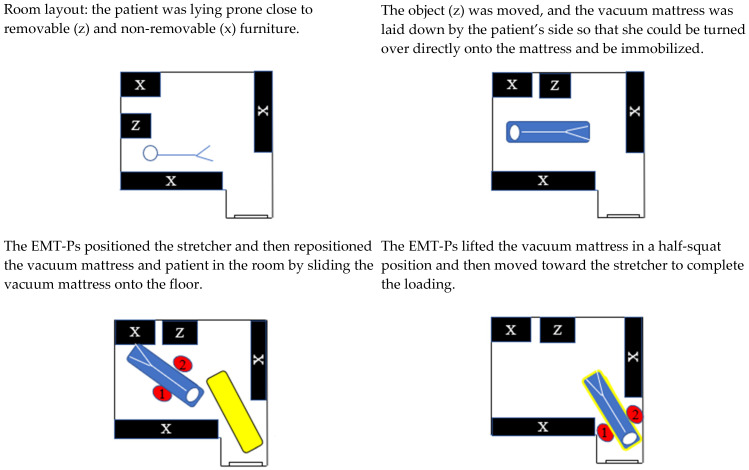
Room layout before the total assistance mode transfer. Black = furniture; Z = removable furniture; X = non-removable furniture; blue = patient on the vacuum mattress; yellow = stretcher; 1 and 2 = EMT-Ps. The red arrows indicate the direction of movement of the EMT-Ps.

Of the nine interventions involving a spinal immobilization where the work area around the patient was limited on arrival, none were considered as a suitable work area for the patient transfer after the preparation task. The EMT-Ps managed the work area by moving objects to another room, moving away from the area (n = 6), or moving the patient (n = 6). In five cases, the EMT-Ps partially cleared the work area, but this was considered insufficient (rated very unfavorable to unfavorable after the preparation subtask); there was a risk of interfering with the EMT-Ps’ movements and postures. For example, in a case where an elderly patient was lying on the floor of a nursing home, the two EMT-Ps prepared the work area by moving objects in the room, reorienting the patient’s body so that her head faced the door, adjusting the height of the stretcher and positioning it close to the patient’s head (Figure 4). Although these preparation operations significantly facilitated the transfer, some objects were still inside the work area. In some cases, the EMT-Ps did not move the patient when it was possible to do so (n = 3), or they could have cleared the work area near the patient or kept it clear but did not (n = 4).

Regarding patient–stretcher configuration, the stretcher’s position in the lateral direction relative to the EMT-Ps and the height adjustment of the stretcher were considered suitable in most cases (8 out of 9 and 7 out of 9, respectively; Table 1). About 57% of the patients were transferred to the stretcher headfirst. When using the vacuum mattress, the feet-first patient orientation was problematic if the stretcher height was not properly adjusted, as the EMT-Ps had to use compensatory movements to free the head end of the stretcher (e.g., raising their shoulders, increasing elbow flexion, and straightening their body by standing on their tiptoes to help lift the foot end of the vacuum mattress).

**Figure 5:**
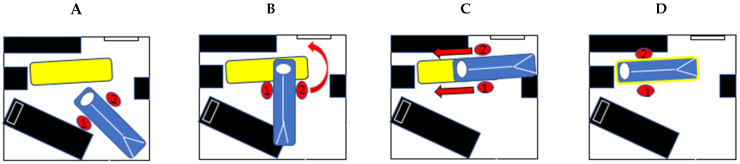
Movements observed in a context of limited space and spinal immobilization indication. (**A**) Limited work area did not allow a favorable alignment at the end of the vacuum mattress. (**B**) The EMT-Ps quickly deposited the head end of the mattress on the foot end of the stretcher and used it as a pivot point to achieve a favorable alignment. (**C**,**D**) The EMT-Ps engaged in the repositioning operation by sliding the vacuum mattress onto the stretcher until it reached the desired position. 1 and 2 = EMT-Ps. The red arrows indicate the direction of movement of the EMT-Ps.

During the transfer subtask, the main postures observed were the squat position during the pickup phase and an upright neutral posture during the loading phase (Table 2). The position of the feet was predominantly facing the load being moved, which limits the level of asymmetry during lifting and lowering. A few cases of desynchronization were noted during lifting or lowering (n = 4), including one case where clear desynchronization was noted during these two phases of the transfer. The EMT-Ps’ hand positions were most often below the knee when lifting the load from the ground, and most EMT-Ps stood erect and extended their elbows to load the patient onto the stretcher (moderate lever arm). A partial loading method, when less than half of the equipment is first placed on the stretcher and then slid onto the stretcher, was observed on only four different occasions. In such a case (Figure 5), the stretcher was positioned less than a meter from the patient’s head during preparation. The patient was transferred to the stretcher headfirst for a partial drop-off with a 90-degree alignment (considered as unfavorable in the grid). The other three transfers with partial loading were made with the patient’s feet first with a 0-degree alignment.

#### 3.2.2. Spinal Immobilization and Suitable Work Area

In 10 situations, the EMT-Ps had the space they needed to install the patients on the equipment and transfer them to the stretcher. In one case, there were objects on the floor in the work area during the transfer (within the one-meter perimeter around the patient). These objects (sheets, a monitor, a survival kit, and an oxygen cylinder) had been placed there during preparation.

The position of the stretcher in the lateral and AP directions, the stretcher alignment, and its height adjustment were all considered suitable in most cases (9 out of 10, 7 out of 10, 8 out of 10, and 9 out of 10, respectively; Table 1). Most transfers to the stretcher were made with the patient’s feet first.

The main postures observed were similar to those observed in a limited workspace (Table 2). No partial loading method was observed. A few cases of desynchronization were noted during lifting or lowering (n = 4), including one case where clear desynchronization was noted during these two phases of the transfer.

#### 3.2.3. Without Spinal Immobilization and with a Limited or Suitable Work Area

For patient transfers without the need for spinal immobilization (Figure 1; transfer families 5 to 8), three patients presented an isolated hip trauma and were transferred onto the stretcher using the scoop. Of the five interventions where the work area around the patient was limited on arrival, one was considered as a suitable work area for the patient transfer after the preparation. In two of five cases, the EMT-Ps partially cleared the work area, but this was considered insufficient. In two other cases, the EMT-Ps did not move the patient or cleared the work area near the patient or kept it clear. The lack of space at one end of the stretcher forced the EMT-Ps to position themselves on the side rather than at the end of the stretcher when loading the scoop onto the stretcher, resulting in the adoption of an asymmetrical position during loading.

For all transfers, with and without a limited work area, the stretcher’s position in the lateral direction relative to the EMT-Ps and its height adjustment were considered suitable in most cases (8 out of 8 and 5 out of 8, respectively; Table 1). However, in most cases, unfavorable stretcher alignment and EMT-Ps’ AP displacement over longer distances were noted regardless of the work area available (Table 1). Half of the EMT-Ps adopted a deep squat position, and the other half used a half-squat position to lift the patient (Table 2). Lifts and travels were generally performed with little asymmetry, whereas half of the deposits were marked by trunk asymmetry.

#### 3.2.4. External Assistance

**Figure 6:**
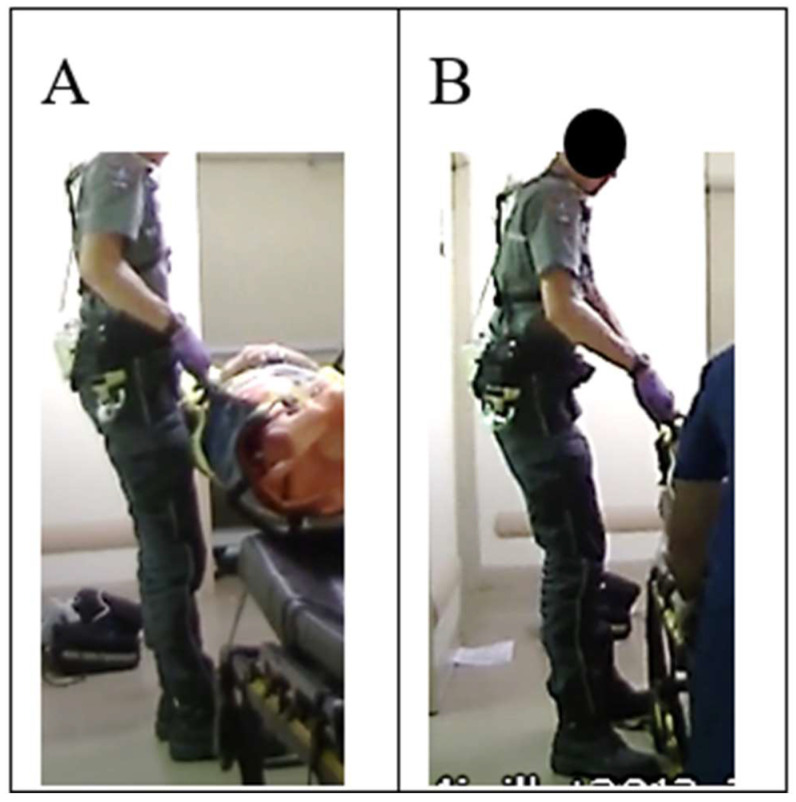
External assistance. EMT-Ps are assisted by a bystander who pushes the stretcher under the vacuum mattress. (**A**) EMT-P’s position observed as the equipment is deposited on the stretcher; (**B**) results of the lever arm as the EMT-P moves backward to avoid being hit by the stretcher.

External assistance was defined as actions such as blocking the stretcher, pushing the stretcher under the transfer equipment, and assisting in the lifting part of the transfer equipment (Supplementary Table S1). Assistance that could cause harm was identified according to the following factors: insufficient speed, stretcher straps getting struck, stretcher hitting EMT-Ps when moved by the helper, and helper letting go of a handle during transfer. Bystanders (patient’s family, neighbors, or other emergency workers, e.g., firefighter, police officer, and EMT-P) provided assistance during transfers in 15 interventions (55.6%). The bystanders helped by lifting a piece of equipment (n = 4), directly lifting the patient (n = 2), blocking the stretcher (n = 3), pushing the stretcher under the vacuum mattress (n = 6), or providing a combination of lifting and blocking or pushing (n = 3). While external assistance was sometimes judged to be helpful (6/15), some problems were observed. For instance, when an external helper pushed the stretcher under the vacuum mattress during the transfer, the bystander had difficulty controlling the stretcher’s direction or speed (4 out of 9); the stretcher straps got stuck in the wheels (2 out of 9); the bystander inadvertently raised the height of the stretcher by pressing its height control button (2 out of 9); or during the push, the stretcher hit the EMT-Ps’ legs or feet because the vacuum mattress and the stretcher were the same width (2 out of 9, Figure 6). Transfers where external assistance was considered at least favorable were generally faster than when there were problematic elements (average duration 4.33 s ± 1.43 s vs. 7.23 s ± 3.48 s). For instance, in two of these cases, the time taken to transfer the patient to the stretcher was almost twice the average observed for all other transfers: 13.2 s (the bystander had difficulty controlling the direction and movement of the stretcher) and 12.9 s (the bystander pressed the stretcher’s height control button while pushing it under the mattress). In most cases, to avoid being hit by the stretcher while holding the vacuum mattress, the EMT-Ps took a step backward (5 out of 6). This maneuver may have the effect of lengthening the lever arm (2 out of 6) (Figure 6).

## 4. Discussion

The objectives of this study were to describe patterns of EMT-Ps’ behaviors during non-mobile patient transfers observed in real-life work situations and to highlight safe work operations during these patient transfers. Observation grids were designed, validated, and used to analyze the video sequences and highlight the operations executed by the EMT-Ps during the preparation subtask, the EMT-Ps’ movements and postures during the transfer subtask, and certain contextual variables observed during the preparation and transfer tasks (work area, type of grip, and availability and quality of external assistance). The results showed that even when stratified based on contextual factors, EMT-Ps’ behaviors during the preparation and transfer subtasks varied greatly between individuals and interventions. While EMT-Ps perform several operations to facilitate patient transfers, these are not always optimal from a safety point of view. Although it might be tempting to believe that the involvement of a third person in the transfer should be helpful, the results suggest that it might actually hinder EMT-Ps’ work in many situations.

When properly executed, the elements of the patient–stretcher configuration have the potential to improve lifting conditions. Among the patient–stretcher configuration elements, the alignment and anterior–posterior distance were the most impacted by a limited work area, especially when transferring a patient with a vacuum mattress. When the workspace was not limited, the most common method observed for positioning the vacuum mattress was to place the stretcher at the end of the vacuum mattress, which requires considerable space: at least 3.7 m. One of the reasons for this is that the location of the handles on the equipment forces the operators to work face to face on the restricted side of the equipment, which encourages lateral movement during the travel phase. The limited working space means EMT-Ps have to adopt different alignments between the stretcher and transfer equipment. For other equipment, such as the backboard and scoop, the EMT-Ps are located at each end, and they grab the equipment’s frame directly. While this type of positioning around these two pieces of equipment may help to minimize the AP distance (e.g., by positioning the equipment and stretcher side by side), this was not observed in most situations. This may have happened because it was not always possible to bring the stretcher close to the patient, or because the EMT-Ps chose not to opt for this alignment between equipment. Suboptimal alignment of the stretcher and the patient forces EMT-Ps to perform forward and/or sideways movements during the moving phase. These extra steps increase the transfer time, leading to prolonged effort and, thus, contributing to cumulative strain on the musculoskeletal system, which can induce fatigue and lead to injuries [30]. Furthermore, these additional movements often force one of the EMT-Ps to walk backward while carrying the patient, putting them at a greater risk of slipping or tripping. Equipment and training that encourage the EMT-P to walk facing forward during patient transport could potentially result in safer transit [31].

Regarding the orientation of the vacuum mattress, it is more likely determined by EMT-Ps’ preference than by the situational factors studied. The feet-first orientation was rated unfavorable in the ergonomic observation grid, as there is a greater likelihood of contact between the stretcher and the mattress when the patient’s feet pass over the head of the stretcher than when the patient’s head passes over the foot of the stretcher. One method of reducing the risk of the vacuum mattress contacting the stretcher is to adjust the height of the stretcher accordingly. This can be done by lowering the stretcher to a height below the level required for a headfirst orientation. The results indicated that a favorable stretcher height adjustment (neither too high nor too low) was a reliable predictor of the absence of compensatory movements during loading, regardless of the equipment. It is important to consider both EMT-Ps’ heights when adjusting the stretcher’s height to ensure that they will both require minimal compensatory movements. This may present a challenge in practice when the difference in height between teammates is pronounced.

In addition to an optimal patient–stretcher configuration, the use of partial loading can potentially decrease the transfer time and efforts. This technique was observed on only a few occasions and only in a restricted workspace. Moreover, the positive effects of partial loading were observed on only one occasion, when the action seemed to have been planned (Figure 5). In other situations, an unexpected event forced the EMT-Ps to engage in partial loading to reposition themselves in relation to the stretcher and then lift once more to reposition the equipment on the stretcher, which involved additional loading. While properly planned partial loading can reduce the loading time, subsequent actions (e.g., pulling) are necessary to reposition the equipment on the stretcher. EMT-Ps typically reposition the mattress by sliding it over the stretcher. Lifting and carrying tasks are often replaced by pulling and pushing tasks in many work situations (e.g., patient handling) to reduce the mechanical load on the body [32,33]. However, it has been shown that push and pull loads equivalent to 30% of body weight or more can be problematic at the lumbar level [34], depending on the friction between the transfer equipment and the stretcher. In this situation, the choice of a material with a low coefficient of friction under the vacuum mattress could help to minimize the friction force when the equipment is slid over a surface. The pros and cons of changing the cover under the mattress must be assessed in light of the overall conditions in which the mattress is used. Further studies are needed to better compare the biomechanical advantages and drawbacks of various transfer and loading methods and help understand the best compromises.

During the pickup phase, EMT-Ps adopt a squat or a semi-squat position in almost all situations, regardless of the contextual factors. The squat is often considered the reference position for lifting heavy loads, as it reduces the moment at the lumbar joints and force generated by low back muscles [35]. For decades, workers involved in manual material handling have been told to “use their legs, not their back” [36]. While this kind of advice has been shown to lead to squat-like behavior (as in the current study), at least in the short term, it often fails to reduce MSD risk [37]. Many factors might limit the effectiveness of such advice. First, the use of large muscle mass requires a substantial expenditure of energy, which can result in fatigue and, consequently, contribute to injury [16]. The half-squat posture is defined by moderate flexion of both knees and the trunk, which enables inter-joint coordination patterns that appear functional in reducing muscular effort [38]. Furthermore, adopting an initial squatting (or half-squat) position does not necessarily mean that individuals are using a less strenuous movement pattern. In a couple of cases, the initial squat position was switched to a stoop position at the instant of lifting. This technique is defined by a sequential movement involving knee flexion during the pickup phase, followed by a fast knee extension (squat to stoop) and then a hip and lumbar extension. In manual handling, this technique is used more often by women and novice handlers [16,39]. According to Maduri et al. [40], the stretching of the passive tissues is a way to transfer the energy to the following contraction during the pickup. Although it seems favorable in terms of force and energy savings, it might also increase the risk of injuries. The effects of this technique when workers are lifting heavier weights, such as those presented in this study, remain unknown. Coordination between the two team members is also crucial to better distribute the load among the team members and avoid sudden efforts. Several situations have been identified as problematic, particularly with regard to synchronization when lifting, transporting, and depositing equipment. A lack of synchronization can result in higher spinal loads on one team member than on the other [41]. Manual handling guidelines recommend that people involved in team lifts be of similar standing height [42]. The results of this study indicated that a lack of synchronization between team members occurred even when their standing heights matched. It is important to remind workers to use a countdown (e.g., “3-2-1-lift” countdown) or some other strategy to synchronize their movement.

When space is limited, EMT-Ps often manage their workspace as best they can. One question is why they do not always organize things favorably when space is not limited. First, the time pressure associated with the patient’s condition may force healthcare personnel to act quickly and use inappropriate postures [43]. When working hastily, they may also become distracted and forget some of the steps in safe handling techniques. The notion of urgency, which is mainly related to the care required by the patient, seems to be very important when EMT-Ps have to choose a compromise, sometimes to the detriment of their own health and safety [5,20]. However, only three patients in this study were evacuated in urgent mode, and the patterns of EMT-Ps’ behaviors did not necessarily differ from those observed in non-urgent contexts. Another possible explanation concerns personal barriers to the use of safe techniques or equipment. Several such barriers to the use of equipment have been identified, such as time constraints, operational difficulties, space restrictions, etc. [44]. Factors favoring the implementation of primary preventive interventions have also been identified, such as motivation, ability, and opportunity [45]. Colleague and employer support, ease of use, and self-efficacy are also on the list of facilitators. Another possible explanation is insufficient training in patient handling. As far as we know, some of these factors, particularly elements of the patient–stretcher configuration, are not explicitly taught, which may explain why EMT-Ps did not apply them. As suggested by other researchers, training on manual material handling should go beyond the instruction to “use the knees” [37]. It should consider the dynamic and changing environment in which the workers operate. The variability of the work situations is often underestimated. Training content could be improved by focusing on EMT-Ps’ ability to adapt to different situations and their work planning skills. Significant motor training based on motor learning principles and techniques, such as video and real-time feedback, should also be used to develop and automatize coordination patterns that reduce biomechanical risk factors for MSDs. Such training has shown great potential for injury prevention by reducing lumbar spine flexion in novice caregivers transferring patients [46].

External assistance has the potential to be beneficial in reducing EMT-Ps’ physical workload. For example, blocking or holding the stretcher during a transfer is a passive type of assistance that had no negative impact. It was helpful if the bystander blocked the stretcher’s mattress at one end to prevent it from sliding off, given that it is lightly attached to the stretcher with Velcro. If the mattress is displaced during a transfer, the EMT-Ps must make an extra repositioning maneuver after loading. The stretcher’s brake, located on the two rear wheels (foot end), could also be used to immobilize the stretcher. On the other hand, in many cases, external assistance resulted in excessive effort, sudden effort, or prolonged cumulative effort, partly due to the bystander’s inexperience or problems with unsecured equipment such as stretcher straps. Thus, unless there are qualified staff members (e.g., EMT-Ps) available to push and direct the movements of the stretcher in an area where space is limited or to help with lifting the vacuum mattress, passive types of help appear to be the preferred kind of assistance and the least likely to cause problems. Particular attention should always be paid to details, such as equipment management (e.g., straps), to avoid sudden exertion, especially when bystanders are invited to participate.

A notable strength of the study is the comprehensive information derived from EMT-Ps’ actual work in different intervention contexts (i.e., 27 different situations involving 27 teams of EMT-Ps). The study also addressed a less well-documented aspect of the job, namely the preparation of patient transfers under real working conditions. Few previous studies have done so in the field [5], while laboratory studies standardize many of these preparation operations [7,8], making it more difficult to identify safe vs. less safe work operations. Similarly, external assistance can only be truly evaluated in a real work situation. The proposed stratification, inspired by previous studies [5,7,8,9], constitutes a strength of this study, enabling the analysis of variability in transfers in accordance with the situational factors identified, and the documentation of work strategies. This may be beneficial to EMT-Ps’ training, the organization of work, and the design of equipment. Another of the study’s strengths lies in the development and utilization of a validated grid that facilitates the identification of the key elements of patient preparation and transfer. The use of this grid could potentially be extended to other transfer contexts involving different types of equipment.

Some limitations were identified in this research. Only one camera was used to capture the interventions, and the focus was on only one of the EMT-Ps, which made it hard to obtain an overall view of the space and of the other EMT-P. The field of view was not constant, and the coming and going of bystanders in front of the camera meant that some variables were harder to observe. One intervention was filmed outside at night and was too dark for all the elements to be analyzed. The analysis was based solely on the video observation. Although post-intervention interviews were conducted, the line of questioning did not allow us to understand the reasons for the EMT-Ps’ actions and decisions. Another limitation is that the student who led the project had been employed as an EMT-P since 2012 (but not by the companies where the observations were carried out), which could have biased the observation and analysis of the situations. This study did not analyze patient positioning on the transfer equipment during the preparation and repositioning subtasks; these subtasks may also reveal exposure to MSD risks. The EMT-Ps were recruited on a voluntary basis. It is possible that the EMT-Ps being observed changed or paid particular attention to the techniques they used to perform their tasks. However, the results of this study show certain recurring incidents and strategies that seem to arise from regular practices of EMT-Ps. Finally, another limitation of the study concerns the “regional” nature of the observations (e.g., equipment used, training received by EMT-Ps, and work organization), which may not be fully replicated in other workplaces elsewhere in the world. Nevertheless, these results illustrate the discrepancy that can sometimes exist between what is prescribed or requested of workers and what actually happens in a work situation, especially when the intervention context is highly variable.

## 5. Conclusions

This research highlighted safe work operations during patient transfers. The organization of the work area appeared to be very important because it predicted the outcome of the subsequent operations. However, elements such as good lateral and anterior–posterior distances between the equipment and the stretcher were favored by a limited work area. The fact that even when there was a suitable work area some elements of the patient–stretcher configuration were still unfavorably executed confirms that ignorance or misunderstanding of safety issues exists in the EMT-P community.

EMT-Ps’ work context is hard to control and adapt; therefore, an emphasis should be placed on training and education. EMT-Ps’ competence is expressed in how they adapt their behaviors according to their evaluation of the various contexts they work in. A certain number of hours are planned annually for clinical updates and training, and some of this time should be dedicated to safe work techniques and how to adapt to the variability of real prehospital work contexts. Future studies should address the enhancement of existing training programs on safe work techniques and assess their efficacy in reducing the workload for preventing work-related low back pain.

## Figures and Tables

**Table 1:** Observation rate for patient–stretcher configuration operations during the preparation subtask (percentage of occurrence in parentheses).

	With Spinal Immobilization (n = 19)				Without Spinal Immobilization (n = 8)				Total (%)	
	Limited Work Area (n = 9)		Suitable Work Area (n = 10)		Limited Work Area (n = 5)		Suitable Work Area (n = 3)			
	F/VF	U/VU	F/VF	U/VU	F/VF	U/VU	F/VF	U/VU	F/VF	U/VU
Align	5 (56%)	4 (44%)	8 (80%)	2 (20%)	1 (20%)	4 (80%)	2 (67%)	1 (33%)	59%	41%
Steer	4 (57%)	3 (43%)	4 (44%)	5 (56%)	2 (100%)	0 (0%)	1 (50%)	1 (50%)	55%	45%
ML position	8 (89%)	1 (11%)	9 (90%)	1 (10%)	5 (100%)	0 (0%)	3 (100%)	0 (0%)	93%	7%
AP position	5 (56%)	4 (44%)	7 (70%)	3 (30%)	1 (20%)	4 (80%)	0 (0%)	3 (33%)	44%	56%
Adjust height	7 (78%)	2 (22%)	9 (90%)	1 (10%)	4 (80%)	1 (20%)	1 (33%)	2 (67%)	78%	22%

F/VF: favorable and very favorable or neutral if no elements in F/VF categories; U/VU: unfavorable or very unfavorable. AP: anterior–posterior; ML: medial–lateral.

**Table 2:** Observation rate for posture and movement elements of the ergonomic grid in the different phases of the transfer subtask (percentage of occurrence in parentheses).

	With Spinal Immobilization		Without Spinal Immobilization	
	Limited work area (n = 9)	Suitable work area (n = 10)	Limited work area (n = 5)	Suitable work area (n = 3)
Whole-body lifting position	Squat (50%) Half squat (50%)	Squat (44%) Half squat (33%)	Squat (40%) Half squat (40%)	Squat (50%) Half squat (50%)
Whole-body loading position	Neutral (67%) Squat (22%)	Neutral (89%) Trunk flexion (11%)	Trunk flexion (75%) Squat (25%)	Neutral (50%) Squat (50%)
Postural asymmetry (pickup)	Neutral (100%)	Neutral (100%)	Neutral (100%)	Neutral (50%) Awkward (50%)
Postural asymmetry (travel)	Neutral (78%) Moderate (22%)	Neutral (56%) Moderate (44%)	Neutral (100%)	Neutral (50%) Moderate (50%)
Postural asymmetry (loading)	Neutral (89%) Awkward (11%)	Neutral (78%) Awkward (11%)	Awkward (50%) Moderate (25%)	Neutral (50%) Awkward (50%)
Feet position (pickup)	Even stance (100%)	Even stance (100%)	Even stance (100%)	Even stance (100%)
Hand position (pickup)	Low (44%) Neutral (33%)	Low (50%) Moderate (40%)	Moderate (44%) Low (44%)	Moderate (100%)
Lever arm (travel)	Short (56%) Large (22%)	Large (57%) Moderate (43%)	Large (60%) Moderate (20%)	Short (100%)
Lever arm (loading)	Moderate (63%) Large (25%)	Moderate (57%) Large (43%)	Moderate (50%) Large (25%)	Large (100%)
Synchronized movements (pickup)	Synchronized (78%) Unsynchronized (22%)	Synchronized (90%) Unsynchronized (10%)	Synchronized (100%)	Synchronized (67%) Unsynchronized (33%)
Synchronized movements (loading)	Synchronized (75%) Unsynchronized (25%)	Synchronized (70%) Unsynchronized (30%)	Synchronized (100%)	Synchronized (100%)
Loading methods	Fast loading (57%) Expected (43%)	Expected (100%)	-	-

## Data Availability

The data supporting the results of this study are part of an institutional database whose management framework is the responsibility of the corresponding author (P.C.). The objective of this database is to enhance the working conditions of EMT-Ps by means of an analysis of their work activity. Requests for access to the database data should be directed to the corresponding author (P.C.).

## Supplementary Materials

The following supporting information can be downloaded at https://www.mdpi.com/article/10.3390/healthcare13060611/s1: Table S1. Ergonomic observation grid, part I. Items from the observation grid covering contextual variables; Table S2. Ergonomic observation grid, part II. Items from the observation grid covering the operations performed during the preparation subtask and the six-level categorization system chosen to assess the MSD risk associated with each item (very unfavorable, unfavorable, neutral, favorable, very favorable, and not applicable or impossible to observe); Table S3. Ergonomic observation grid, part III. Items from the observation grid covering the movements performed during the transfer subtask; Table S4. Gwet’s AC1 for operations, movements and postures, and contextual variables.
