# Supplementary material for: Paramedics’ Behavior Patterns When Transferring Non-Mobile Patients from the Ground to a Stretcher
Source: Healthcare (Basel). 2025 Mar 12;13(6):611. doi: 10.3390/healthcare13060611 (PMC11942343; doi:10.3390/healthcare13060611)
Supplement: Supplementary file 1 [file healthcare-13-00611-s001.zip › Supplementary Materials.pdf]

Supplementary Materials

Table S1. Ergonomic observation grid, part I. Items from the observation grid covering contextual variables.

**Contextual variable**	**Observable elements and rating**			
Level of interference in the work area close to the patient at the start of the pickup phase	Very limited: 3 or 4 zones around the patient do not have a clear space of 1 m around the patient or height space limitation.	Limited: 2 zones do not have a clear space of 1 m around the patient.	Few limitations: 1 m free space in 3 zones around the patient.	Suitable work area: 1 m available around the patient in all 4 zones.*
External assistance	Blocking the stretcher.	Pushing the stretcher under the transfer equipment.	Lifting part of the transfer equipment.	
Quality of the external assistance:	Problems related to external help+ (*e.g., problems occurring when the stretcher was pushed by a helper: straps jammed in the wheels, difficulty controlling the stretcher's direction or speed, unintentional raising of the stretcher's height by pressing the height control button, the stretcher hit the EMT-Ps' legs or feet*).	No problems observed.		

* Stretcher positioned at less than 1 m is not considered to interfere. + Assistance that can cause harm has been identified according to the following factors: insufficient speed, stretcher straps get struck, stretcher hits EMP-Ts when moved by the helper, helper lets go of a handle during transfer. A bystander might still interfere with the transfer even if they do not participate.

Table S2. Ergonomic observation grid, part II. Items from the observation grid covering the operations performed during the preparation subtask, and the six-level categorization system chosen to assess the MSD risk associated with each item (very unfavorable, unfavorable, neutral, favorable, very favorable and not applicable or impossible to observe).

**Preparation subtask:** From the arrival on scene to the transfer phase.						
**Operations**	**Very unfavorable**	**Unfavorable**	**Neutral**	**Favorable**	**Very favorable**	**Not assessable or not applicable**
Move interfering objects in workspace.<br><br>(*The EMT-Ps' equipment can be considered as interfering objects.*)	None of the zones occupied by interfering and removable objects are cleared.<br>Objects are not moved or are moved but remain within the workspace.<br>An object is stepped over.	Some zones are completely free of interfering objects, while at least one zone is still constrained.			All zones are free of interfering and removable objects.	There are no interfering and removable objects in the 4 zones of the workspace.
Move the patient on the floor to create space around the patient.<br><br>(*This does not include changing the patient's position, e.g., from prone to supine.*)	The patient is not moved even though there is at least 2 m of space in the head or foot zone and/or in a lateral zone.<br>Moreover, moving the patient could have improved the available working space.	The patient is moved when there is at least 2 m in the head or foot area and/or in a lateral area, but not enough to clear the working areas, although the working space allowed it.		The patient is moved without all the zones being cleared, and without the possibility of doing more.	The patient is moved to completely clear at least one zone or to improve the patient-stretcher configuration (orientation, alignment, lateral distance, anterior-posterior distance).	The workspace is cleared from the outset in at least 3 zones around the patient or there is not enough space to move the patient.
Adjust stretcher height	Inappropriate height adjustment	When both EMT-Ps are seen		When only one EMT-P is	Minimal compensatory	

(*Observed when the transfer equipment arrives close to the stretcher, before loading, as it may cause obvious movement compensation: shoulder elevation, elbow flexion, lateral trunk flexion or standing on tiptoe when too high, or sagittal trunk flexion when too low. It may also cause minimal movement compensation such as minimal trunk flexion, knee flexion, elbow extension.)*	(compensatory movement evident during loading for both EMT-Ps).	on video images, minimal compensatory movement by one EMT-P and obvious compensatory movement by the other EMT-P. When only one EMT-P is visible on the video images, obvious compensatory movement of the visible EMT-P.		visible on the video images, minimal compensatory movement of the visible EMT-P.	movement for both EMT-Ps.	
Position the stretcher close to the patient (or vice versa) at an optimal lateral distance.<br><br>(*Reference distance: length of the vacuum mattress.*)	There is at least the length of a vacuum mattress between the transfer equipment and the stretcher.	There is at least half the length of a vacuum mattress (maximum one vacuum mattress) between the transfer equipment and the stretcher.			There is less than half the length of a vacuum mattress between the transfer equipment and the stretcher.	
Position the stretcher close to the patient (or vice versa) at an optimal anterior-posterior distance.	EMT-Ps step forward/backward.		No forward/ backward steps.			

Align the stretcher in relation to the patient's longitudinal axis (or vice versa).<br><br>(*Angle between the transfer equipment and the stretcher.*)	Stretcher axis offset greater than 45˚	Slightly misaligned between 10˚ and 45˚			Good alignment or negligible misalignment from approximately 0˚ to 10˚.	
Steer the patient when loading on the stretcher.<br><br>(*Does not apply to scoop and back board)*	Patient's feet first toward the stretcher.				Patient's head first toward the stretcher.<br><br>Rescue seat and using clothes: patient's buttocks first.	Scoop/back board

Table S3. Ergonomic observation grid, part III. Items from the observation grid covering the movements performed during the transfer subtask.

**Movements and postures**	**Phase**			
EMT-P lifting position	Pickup	**STOOP**<br>(*Little to no knee flexion, thoracic portion tends to round at the beginning of the movement.*)	**HALF-SQUAT**<br>(*Less knee flexion than squat, buttocks are higher than knees, shoulders are above or in front of the knees.*)	**SQUAT**<br>(*Thighs are almost parallel to the ground, buttocks are at or lower than knee level.*)
EMT-P loading position	Loading	**TRUNK FLEXION**<br>Sagittal trunk flexion or lateral trunk flexion.	**SQUAT**	**NEUTRAL**<br>Minimal movement.<br>(*Movement performed with full elbow extension.*)
EMT-P postural asymmetry	Pickup<br>Travel<br>Loading	**AWKWARD**<br>Hands are not centered on the body.<br>(*One hand exceeds the midline of the body or at least two of the following: lateral trunk flexion, abduction of one shoulder and adduction of the other, axial rotation of the trunk.*)	**MODERATE**<br>Hands are centered with respect to the hips, but hips are rotated axially with respect to the rest of the body.<br>(*May also include lateral trunk flexion OR one shoulder in abduction and the other in adduction OR trunk rotation.*)	**NEUTRAL**<br>Hands are centered compared to the hips.<br>(*Shoulders and hips are facing the load.*)
Foot position	Pickup<br>Loading	**PARALLEL**<br>Parallel to the equipment.	**ONE FOOT FORWARD**<br>One foot in front of the other. (*Lunge position or when one foot does not touch the ground completely.*)	**EVEN STANCE**<br>Both feet on the ground are facing the load.
Hand position (vertical)	Pickup	**LOW**<br>At knee level or below.	**MODERATE**	**NEUTRAL**

			Between knee and mid-thigh.	Between mid-thigh and waist.
Lever arm (horizontal)	Travel Loading	**LARGE** Equal to or longer than the length of the forearm.	**MODERATE** Shorter than half the length of the forearm.	**SHORT** Hands are very close to the body.
Synchronized movements	Pickup Loading	**UNSYNCHRONIZED** One side or end of the equipment leaves the ground or touches the stretcher before the other.	**SYNCHRONIZED** The equipment leaves the ground and touches the stretcher as one block.	
Loading methods	Loading	**EXPECTED** More than three-quarters of the transfer equipment is first placed on the stretcher.	**HALF LOADING** More than half of the equipment is first placed on the stretcher.	**PARTIAL LOADING** Less than half of the equipment is first placed on the stretcher.

Table S4. Gwet's AC1 for operations, movements and postures, and contextual variables.

	**Intra-reliability**	**Inter-reliability**
**Preparation subtask – Operations**		
Move interfering objects	0.80	0.40
Move the patient	0.50	0.60
Adjust stretcher's height	0.70	0.50
Position the stretcher (ML distance)	1	0.60
Position the stretcher (AP distance)	0.90	0.80
Align the stretcher	1	0.80
Steer the patient	0.90	1
**Transfer subtask – Postures and movements**		
Whole body lifting position	0.70	0.70
Whole body loading position	0.70	0.30
Postural asymmetry (pickup)	1	1
Postural asymmetry (travel)	0.70	0.70
Postural asymmetry (loading)	0.90	1
Feet position (pickup)	0.90	0.90
Feet position (loading)	0.70	0.90
Hand position (pickup)	0.70	0.50
Lever arm (travel)	0.60	0.50
Lever arm (loading)	0.80	0.50
Synchronized movements (pickup)	0.90	0.80
Synchronized movements (loading)	0.80	0.70
Loading methods	1	0.80
**Contextual variables**		
Limited work area at the time of pickup	0.8	0.6
External assistance	1	0.70
Quality of external assistance	0.80	0.80
Total (mean / median)	0.81 / 0.80	0.70 / 0.70
